# Human antibody recognition and neutralization mode on the NTD and RBD domains of SARS-CoV-2 spike protein

**DOI:** 10.1038/s41598-022-24730-4

**Published:** 2022-11-22

**Authors:** Ryota Otsubo, Takeharu Minamitani, Kouji Kobiyama, Junso Fujita, Toshihiro Ito, Shiori Ueno, Itsuki Anzai, Hiroki Tanino, Hiroshi Aoyama, Yoshiharu Matsuura, Keiichi Namba, Ken-Ichi Imadome, Ken J. Ishii, Kouhei Tsumoto, Wataru Kamitani, Teruhito Yasui

**Affiliations:** 1 Laboratory of Infectious Diseases and Immunity, Center for Vaccine and Adjuvant Research (CVAR), National Institutes of Biomedical Innovation, Health and Nutrition (NIBIOHN), 7-6-8 Saito-Asagi, Ibaraki, Osaka 567-0085 Japan; 2 Division of Vaccine Science, Department of Microbiology and Immunology, The Institute of Medical Science, The University of Tokyo, 4-6-1 Shirokanedai, Minato-ku, Tokyo, 108-8639 Japan; 3 Laboratory of Adjuvant Innovation, CVAR, NIBIOHN, 7-6-8 Saito-Asagi, Ibaraki, Osaka 567-0085 Japan; 4 Graduate School of Frontier Biosciences, Osaka University, 1-3 Yamadaoka, Suita, Osaka 565-0871 Japan; 5 Graduate School of Pharmaceutical Sciences, Osaka University, 1-6 Yamadaoka, Suita, Osaka 565-0871 Japan; 6 Laboratory of Proteome Research, NIBIOHN, 7-6-8 Saito-Asagi, Ibaraki, Osaka 567-0085 Japan; 7 Department of Infectious Diseases and Host Defense, Graduate School of Medicine, Gunma University, 3-39-22 Syowa-cho, Maebashi, Gunma 371-8511 Japan; 8 Department of Molecular Virology, Research Institute for Microbial Diseases, Osaka University, 3-1 Yamadaoka, Suita, Osaka 565-0871 Japan; 9 Centre for Infectious Disease Education and Research, Osaka University, 2-8 Yamadaoka, Suita, Osaka 565-0871 Japan; 10 Laboratory of Virus Control, Research Institute for Microbial Diseases, Osaka University, 3-1 Yamadaoka, Suita, Osaka 565-0871 Japan; 11 JEOL YOKOGUSHI Research Alliance Laboratories, Osaka University, 1-3 Yamadaoka, Suita, Osaka 565-0871 Japan; 12 RIKEN SPring-8 Center, 1-3 Yamadaoka, Suita, Osaka 565-0871 Japan; 13 Department of Advanced Medicine for Infections, National Center for Child Health and Development (NCCHD), 2-10-1 Okura, Setagaya-ku, Tokyo, 157-8535 Japan; 14 Center for Drug Discovery Research (CDDR), National Institutes of Biomedical Innovation, Health and Nutrition (NIBIOHN), 7-6-8 Saito-Asagi, Ibaraki, Osaka 567-0085 Japan; 15 Department of Bioengineering, School of Engineering, The University of Tokyo, 7-3-1 Hongo, Bunkyo-ku, Tokyo, 113-8656 Japan; 16 Medical Proteomics Laboratory, The Institute of Medical Science, The University of Tokyo, 4-6-1 Shirokanedai, Minato-ku, Tokyo, 108-8639 Japan; 17 Present Address: Toyama Prefectural Institute for Pharmaceutical Research, 17-1 Nakataikoyama, Imizu, Toyama 939-0363 Japan; 18 Present Address: Laboratory of Experimental Immunology, Department of Regeneration Science and Engineering, Institute for Life and Medical Sciences, Kyoto University, 53 Shogoin Kawahara-cho, Sakyo-ku, Kyoto, 606-8507 Japan

**Keywords:** Viral infection, SARS-CoV-2, Antibodies

## Abstract

Severe acute respiratory syndrome coronavirus 2 (SARS-CoV-2) causes coronavirus disease 2019 (COVID-19). Variants of concern (VOCs) such as Delta and Omicron have developed, which continue to spread the pandemic. It has been reported that these VOCs reduce vaccine efficacy and evade many neutralizing monoclonal antibodies (mAbs) that target the receptor binding domain (RBD) of the glycosylated spike (S) protein, which consists of the S1 and S2 subunits. Therefore, identification of optimal target regions is required to obtain neutralizing antibodies that can counter VOCs. Such regions have not been identified to date. We obtained 2 mAbs, NIBIC-71 and 7G7, using peripheral blood mononuclear cells derived from volunteers who recovered from COVID-19. Both mAbs had neutralizing activity against wild-type SARS-CoV-2 and Delta, but not Omicron. NIBIC-71 binds to the RBD, whereas 7G7 recognizes the N-terminal domain of the S1. In particular, 7G7 inhibited S1/S2 cleavage but not the interaction between the S protein and angiotensin-converting enzyme 2; it suppressed viral entry. Thus, the efficacy of a neutralizing mAb targeting inhibition of S1/2 cleavage was demonstrated. These results suggest that neutralizing mAbs targeting blockade of S1/S2 cleavage are likely to be cross-reactive against various VOCs.

## Introduction

Severe acute respiratory syndrome coronavirus 2 (SARS-CoV-2) is the etiological agent of coronavirus disease 2019 (COVID-19). It belongs to the subgenus Sarbecovirus and has approximately 80% homology with the genome of SARS-CoV^1^. Substantial numbers of patients with COVID-19 have severe respiratory symptoms, sometimes exacerbated by sepsis^2^. Although COVID-19 is associated with lower mortality than SARS-CoV infection, human-to-human transmission of SARS-CoV-2 is highly efficient^3^. In SARS-CoV-2 infection, the glycosylated homotrimeric S protein is used for viral entry and membrane fusion^4^. The S protein consists of S1, which contains the N-terminal domain (NTD), the receptor binding domain (RBD) and subdomain (SD), and S2 subunit. The S protein binds to angiotensin-converting enzyme 2 (ACE2) on host cells via the RBD, leading to cleavage of the S protein at the S1/S2 and S2′ sites by cellular protease such as furin and transmembrane serine protease 2 (TMPRSS2) ^5,6^. Once cleaved, S2 promotes virus–host membrane fusion and viral entry.

Almost all of previously reported SRAS-CoV-2 neutralizing monoclonal antibodies (mAbs) target the S protein^4,7,8^. Most of them recognize the RBD and thereby inhibit viral entry into host cells by blocking S protein–ACE2 binding. By contrast, few neutralizing mAbs target the NTD^9,10^. Some mAbs targeting the NTD interact with the S protein, structurally open the RBD, and promote S protein–ACE2 binding, resulting in antibody-dependent enhancement (ADE)^11^.

SARS-CoV-2 variants have emerged as the COVID-19 pandemic continues to spread^12^. In particular, the World Health Organization (WHO) has issued an alert for the spread of Delta and Omicron as variants of concerns (VOCs)^13^. These VOCs contain numerous mutations in the S protein and other proteins and more readily evade antibodies produced by humoral immunity than wild-type (WT) SARS-CoV-2^14^. Therefore, neutralizing mAbs with cross-reactivity are required.

Previous studies of neutralizing mAbs have demonstrated that inhibiting the interaction between the S protein and ACE2 suppresses SARS-CoV-2 infection. However, there are none reports of suppressing infection by dampening S1/2 cleavage. In this study, we isolated 2 neutralizing mAbs, NIBIC-71 and 7G7, with different mechanisms using our method with memory B cells immortalized with Epstein-Barr virus (EBV). The neutralizing activity of these mAbs was evaluated in vitro and in vivo. We clarified their mechanisms of neutralization.

## Results

### Isolation of 2 neutralizing mAbs, NIBIC-71 and 7G7

**Figure 1:**
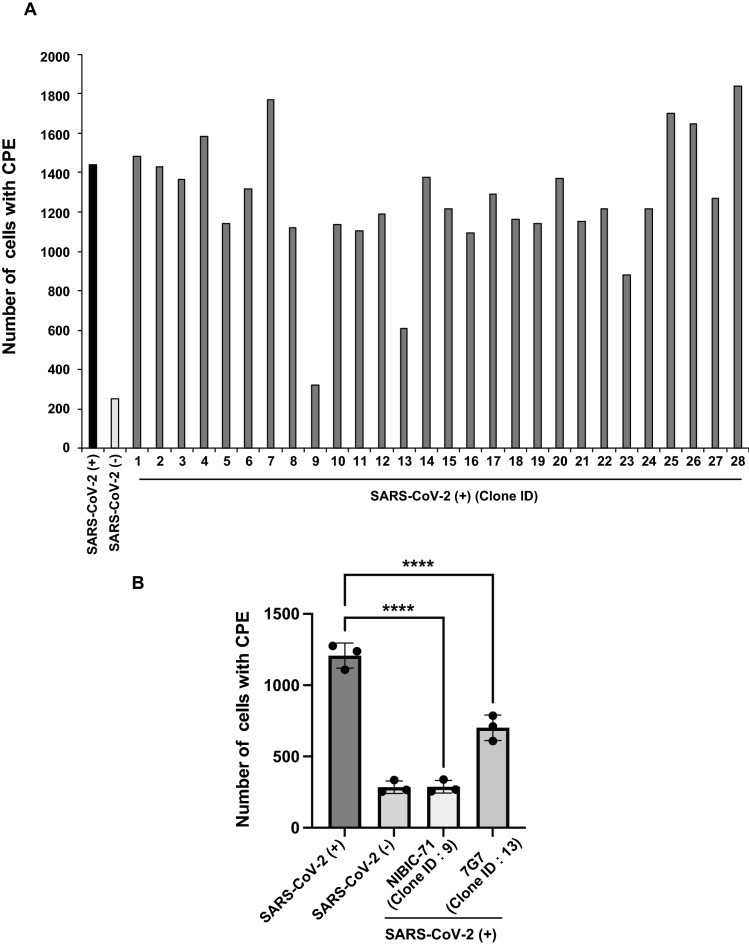
Two LCL supernatants suppressed CPE due to SARS-CoV-2 infection. (**A**) The number of cells with CPE in each panel of Fig. S1. (**B**) Two LCL supernatant (Clone ID: 9 and 13) were incubated with or without WT SARS-CoV-2 for 1 h. VeroE6-TMPRSS2 cells were infected with WT SARS-CoV-2 for 4 days in triplicate, which induced CPE. The number of cells with CPE was counted with ImageJ software. ****P < 0.001 (ANOVA).

In order to obtain anti–SARS-CoV-2 neutralizing human mAbs, peripheral blood mononuclear cells (PBMCs) were isolated from 2 donors who recovered from COVID-19. B cells were infected with EBV, inducing transformation to lymphoblastoid cell lines (LCLs) that can produce antibodies. To select LCLs that secrete antibodies with specificity for the S protein, we performed enzyme-Linked Immuno-Sorbent Assay (ELISA) against the S protein, which identified 28 LCL supernatants with reactivity to the S protein. Furthermore, to evaluate whether each candidate has neutralizing activity or not, cytopathic effect (CPE) inhibitory assays were performed. Two LCL supernatants (clone ID: 9 and 13) inhibited the CPE associated with SARS-CoV-2 infection (Fig. 1A and S1) and then were reproducibly effective (Fig. 1B). Since it is necessary to identify the sequences of S protein–specific mAbs in both LCL supernatants, we carried out B cell receptor (BCR) amplicon sequencing and antibody amino acid sequencing with liquid chromatography/mass spectrometry (LCMS). The sequence information was integrated; we determined the DNA sequences of 2 S protein–specific mAbs, NIBIC-71 and 7G7. Somatic hypermutation (SHM) rates were calculated by comparing DNA sequences of the heavy chain variable region (VH) and the light chain variable region (VL) to germline sequences. As shown in Table 1, NIBIC-71 was derived from IgG1/Igκ with an IGHV3-53 and IGKV1-9 genes, while 7G7 (7G7/λ) was derived from IgG1/Igλ2 with an IGHV3-33 and IGLV3-25 genes. These results demonstrated that our method yielded neutralizing mAbs against emerging infectious diseases.

**Table 1:** Gene usage and CDR3 sequence of NIBIC-71 and 7G7.

mAb	VH	% of identical nucleotides	CDR3	VL	% of identical nucleotides
NIBIC-71	IGHV3-53	96.2	ARDLGPYGVDV	IGKV1-9	97.9
7G7	IGHV3-33	95.6	ARDQGFGDNYYYYGMDV	IGLV3-25	98.2

Each mAb was compared to germline DNA and amino acid sequences. mAb, monoclonal antibody; VH, variable region of the heavy chain; VL, variable region of the light chain; CDR, complementarity determining region; nt: nucleotide.

### Characterization of NIBIC-71, and 7G7

**Figure 2:**
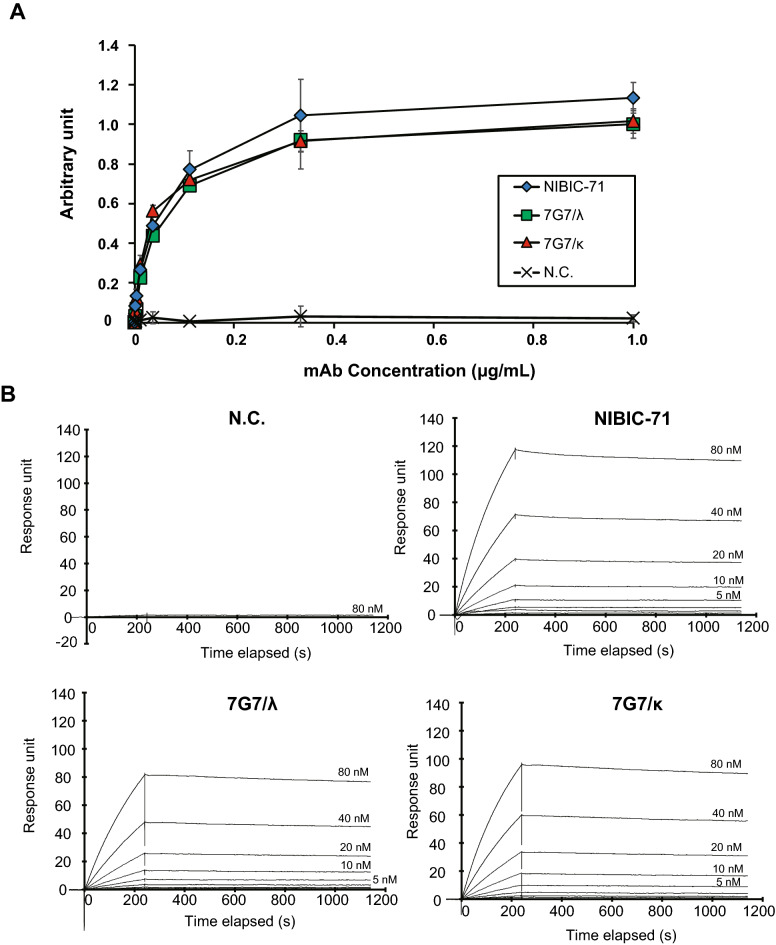
Binding affinity of NIBIC-71 and 7G7 to the S protein. (**A**) The ability to bind to the S protein was evaluated with ELISA and serially diluted recombinant mAbs from 10 to 0.001 µg/mL in triplicate. Blue diamonds indicate NIBIC-71, green squares indicate 7G7/λ, red triangles indicate 7G7/κ, and cross markses indicate negative control mAb (N.C.). (**B**) SPR analyses for these mAbs. The SPR sensorgrams were obtained by injecting twofold serial dilutions of the S protein ranging from 80 to 0.625 nM for NIBIC-71, 7G7/λ, and 7G7/κ. Contact time and dissociation time were set as 240 s and 900 s, respectively. The kinetic values are shown in Table 2.

To characterize NIBIC-71 and 7G7, we prepared recombinant mAbs NIBIC-71, 7G7/λ, and 7G7/κ by replacing the Igλ2 constant region of 7G7/λ with that of Igκ to increase the yield of purified mAb. The S protein–binding ability of these mAbs was assessed in a mAb concentration–dependent manner using ELISA. The half maximal effective concentrations (EC50s) of NIBIC-71, 7G7/λ, and 7G7/κ were comparable (Fig. 2A). In addition, we measured the affinity between each mAb and the S protein using surface plasmon resonance (SPR) analysis. These mAbs indicated a mAb dose–dependent response against the S protein (Fig. 2B). This result implied that these mAb are specific for the S protein. As shown in Table 2, the dissociation constant KD values were, in increasing order, NIBIC-71, 7G7/κ, and 7G7/λ. Taken together, 7G7/κ (IgG1/Igκ) had higher affinity for the S protein than 7G7/λ (IgG1/Igλ2).

**Table 2:** Kinetic parameters from SPR analysis.

mAb	*k*_a_ (1/Ms)	*k*_d_ (1/s)	*K*_D_ (nM)
N.C	7.25 × 10^4^	1.44 × 10^−7^	1.98 × 10^−3^
NIBIC-71	4.77 × 10^4^	6.35 × 10^−5^	1.33
7G7/λ	3.64 × 10^4^	7.39 × 10^−5^	2.04
7G7/κ	5.31 × 10^4^	8.20 × 10^−5^	1.55

N.C. indicated the negative control antibody which have no specificity for the S protein. SPR: surface plasmon resonance, mAb: monoclonal antibody, ka: association rate constant, kd: dissociation rate constant, KD: dissociation constant.

### Binding mode and inhibitory mechanism of NIBIC-71 and 7G7

**Figure 3:**
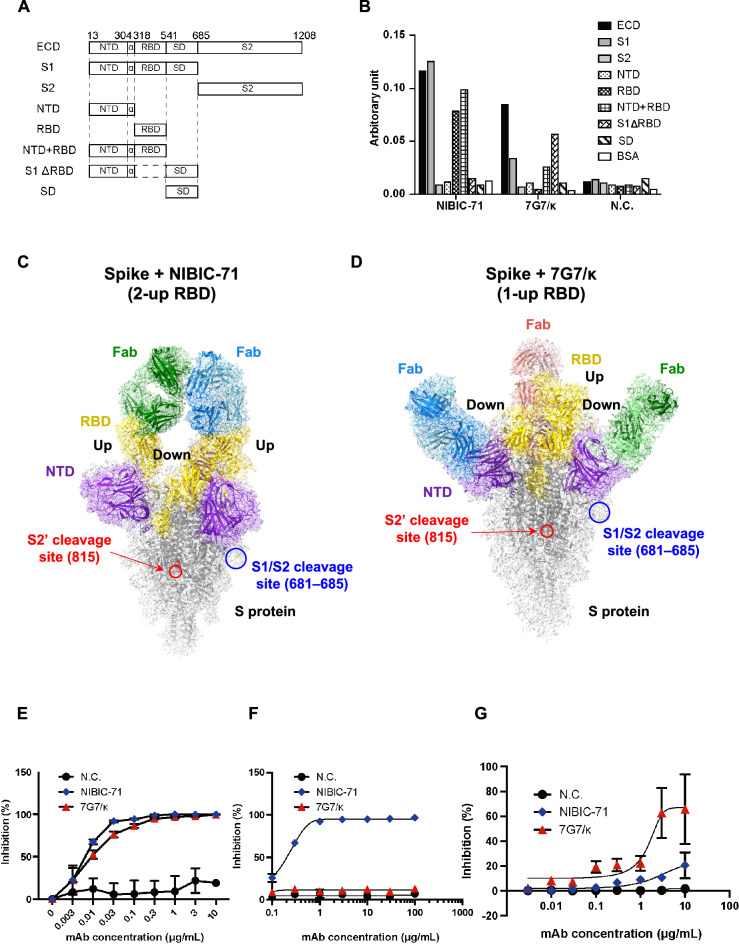
Neutralization mechanisms of NIBIC-71 and 7G7. (**A**) Truncated S proteins tagged with Flag and 6 × His were synthesized with the Bac-to-Bac baculovirus system. Purified proteins were dialyzed with PBS. (**B**) The binding regions of these mAbs were determined using ELISA with the recombinant proteins shown in A as antigens. The vertical axis shows normalized values (arbitrary unit) relative to absorbance obtained with ELISA against His. (**C**) and (**D**) Cryo-EM sharpened maps of the homotrimeric S protein complexed with NIBIC-71 (**C**) or 7G7/κ (**D**). The atomic model of S protein and the homology models of the Fabs are manually fitted into the maps. The regions corresponding to the two mAbs are shown in blue, green, and red. The heavy and light chains are shown in different colors. The NTD and RBD region in the S protein are shown in purple and yellow, respectively. One of S2’ and S1/S2 cleavage site are shown in red and blue circle, respectively. (**E**) The neutralizing activity of these mAbs was assessed with a luciferase assay in triplicate. VeroE6-TMRPSS2 cells were infected with WT SARS-CoV-2 introduced by nanoLuciferase. The cells were pre-incubated with or without serially diluted recombinant mAbs from 10 to 0.001 µg/mL. Bioluminescence was measured using a luminometer. (**F**) The inhibitory effect of S protein–ACE2 binding was evaluated with ELISA in triplicate. (**G**) The inhibitory effect of S1/S2 cleavage was evaluated with ELISA. In E–G, inhibition efficiency (%) was calculated relative to values derived from the negative control samples without any mAbs. ECD, extracellular domain; S1, subunit 1; S2, subunit 2; NTD, N-terminal domain; RBD, receptor binding domain; SD, subdomain.

A series of truncated S proteins were prepared to determine which parts of the S protein were recognized as epitope by NIBIC-71 and 7G7/κ (Fig. 3A). Interactions between each individual truncated protein and each mAb were evaluated using ELISA. NIBIC-71 targeted the RBD. By contrast, 7G7/κ was able to bind to the S1, NTD + RBD, and S1 RBD-deletion mutant (ΔRBD) but not the NTD, SD, or S2 (Fig. 3B). To visualize the binding modes of NIBIC-71 and 7G7/κ, we performed cryo-electron microscopy (cryo-EM) single particle analyses of the S protein complexed with the two mAbs (Fig. S2A–D). Only the densities corresponding to the fragment antigen-binding region (Fab) of each mAb were observed, and we could not build accurate atomic models due to the low local resolution of the Fab region (Fig. S2E,F). The overall map resolution reached 2.64 Å and 2.96 Å for NIBIC-71 and 7G7/κ complexes, respectively (Fig. S2G,H and Table 3). The results illustrated that 2 NIBIC-71 Fabs interacted with 2 Up-RBDs of the homotrimeric S protein (Fig. 3C), while each of three 7G7/κ Fabs binds to each NTD of the homotrimeric S protein (Fig. 3D). In order to assess the inhibitory effect of NIBIC-71 and 7G7/κ on virus entry into host cells, in vitro neutralizing activity assays were performed using luciferase technology. NIBIC-71 and 7G7/κ inhibited viral entry into host cells in a mAb dose–dependent manner (Fig. 3E). In addition, NIBIC-71 had a higher half maximal inhibitory concentration (IC50) than 7G7/κ (Fig. 3E). To clarify how NIBIC-71 and 7G7/κ block SARS-CoV-2 entry into host cells, we first investigated the blockade of S protein–ACE2 binding by NIBIC-71 and 7G7/κ. NIBIC-71 blocked binding, but 7G7/κ did not (Fig. 3F). We next evaluated suppression of S1/S2 cleavage by these 2 antibodies. We demonstrated that 7G7/κ suppresses S1/S2 cleavage but NIBIC-71 does not (Fig. 3G). In conclusion, NIBIC-71 and 7G7/κ suppressed viral entry via different mechanisms. NIBIC-71 targeted the RBD to inhibit S protein–ACE2 binding and 7G7/κ recognized the NTD and somehow dampened S1/S2 cleavage.

**Table 3:** Cryo-EM data collection and image processing.

Dataset	Spike + NIBIC-71	Spike + 7G7/κ
	(2-up RBD)	(1-up RBD)
EMDB accession no	EMD-34031	EMD-34032
Magnification	60,000	60,000
Voltage (kV)	300	300
Electron exposure (e^−^/Å^2^)	60	60
No. of frames per movie	60	60
Defocus range (mm)	− 0.5 to − 2.0	− 0.5 to − 2.0
Pixel size (Å)	1.045	1.287
Symmetry imposed	C1	C1
Micrographs used (no.)	6,603	5,097
Optics group (no.)	14	10
Initial particle images (no.)	1,012,450	645,722
Final particle images (no.)	165,958	140,163
Map resolution (Å)	2.64	2.96
FSC threshold	0.143	0.143
Map resolution range (Å)	2.30–9.65	2.57–16.0

### Neutralizing activity of NIBIC-71 and 7G7 in vivo

**Figure 4:**
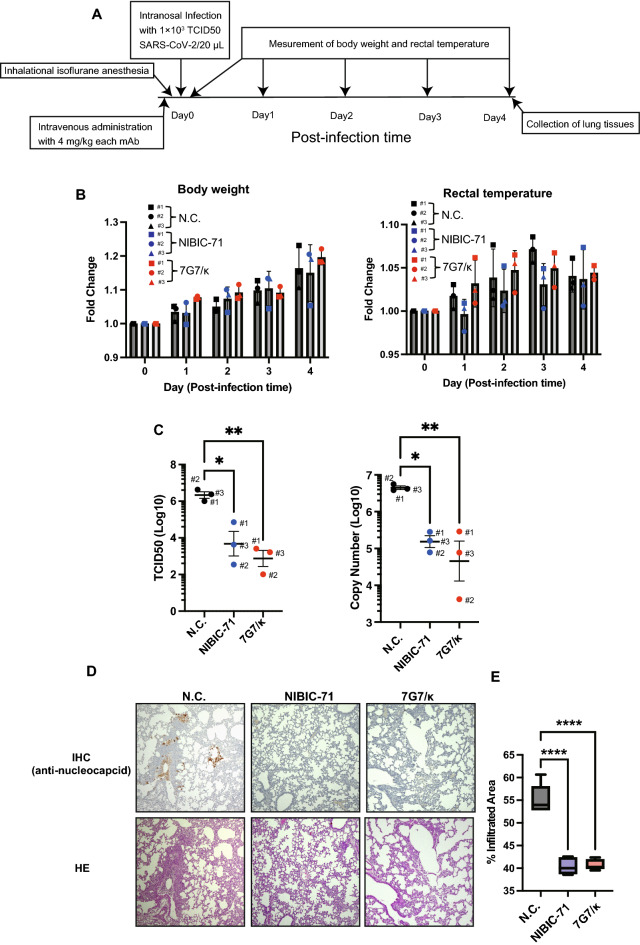
Analysis of in vivo neutralizing activity of NIBIC-71 and 7G7. Male 3-week-old Syrian hamsters that received 4 mg/kg of recombinant mAbs intravenously were infected with WT SARS-CoV-2 via the nasal route. (**A**) the schematic of in vivo infectious experiment. (**B**) Body weight (left) and rectal temperature (right) were monitored up to 4 days after infection (n = 3). Fold change is the value relative to before infection. (**C**) Viral load in the lung at 4 days after infection was shown as the means ± standard error of the mean for TCID50 (left) and copy number (right) (n = 3). (**D**) Histological analysis with IHC (N antigen) (upper) and HE staining (lower). SARS-CoV-2 was detected with an anti-nucleocapsid protein antibody. The brown staining indicated nucleocapsid (N antigens) of SARS-CoV-2. E. The proportion of the area with cellular infiltration was presented as mean ± standard error of the mean^15^. This value is the ratio of the HE-stained area to the whole area. These areas were obtained in 5 sections from each hamster. *P < 0.05; **P < 0.01, ****P < 0.001 (ANOVA). TCID50, tissue culture infectious dose.

We assessed the in vivo neutralizing activity of NIBIC-71 and 7G7/κ against SARS-CoV-2. Syrian hamsters were infected with WT SARS-CoV-2 via the nasal route after intravenous injection of NIBIC-71, 7G7/κ, or negative control mAb. Each hamster’s body weight and rectal temperature were monitored every day until 4 days post-infection (Fig. 4A). There were no significant differences between mAb groups with respect to changes in body weight and rectal temperature (Fig. 4B). Moreover, lung tissue was collected from hamsters infected at 4 days post-infection. Viral load in the lung was measured using the CPE assay and reverse transcription (RT)-PCR. As shown in Fig. 4C, NIBIC-71 and 7G7/κ reduced viral loads significantly more than the negative control mAb. Furthermore, we examined how much tissue damage caused by SARS-CoV-2 infection was alleviated by each mAb with histological analysis of lung tissue sections. SARS-CoV-2 nucleocapsid protein was detected in the lung sections of hamsters treated with the negative control mAb but not detected in hamsters treated with either NIBIC-71 or 7G7/κ mAb (Fig. 4D). In addition, we assessed the area of lung cellular infiltration including epithelial cells using lung tissue sections with hematoxylin and eosin (HE) staining. The NIBIC-71 and 7G7/κ groups had much smaller cellular infiltration areas than the negative control mAb group (Fig. 4E). These findings implied that these mAbs suppressed inflammation caused by SARS-CoV-2 infection. Taken together, these results suggested that NIBIC-71 and 7G7/κ had neutralizing activity in vivo and did not induce ADE.

### NIBIC-71 and 7G7 lost neutralizing activity against the Omicron variant

**Figure 5:**
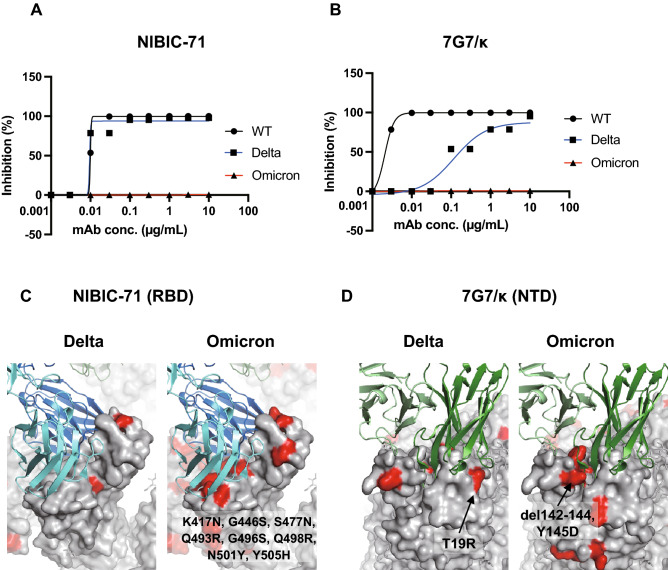
Evaluation of in vitro neutralizing activity against the Delta and Omicron variants. (**A**) and (**B**) VeroE6-TMPRSS2 cells were infected with WT SARS-CoV-2, Delta, and Omicron with or without serially diluted NIBIC-71 (**A**) and 7G7/κ (**B**) from 10 to 0.001 µg/mL in quadruplicate. The number of wells with CPE was counted. Viral loads were presented as TCID50s. Inhibition efficiency (%) was calculated relative to values derived from the negative control samples without any mAbs. (**C**) and (**D**) Binding region of NIBIC-71 (**C**) and 7G7/κ (**D**). Homology models of the two Fabs are generated and manually fitted into the cryo-EM maps. Surfaces of the S proteins are shown in gray, and the mutation points in Delta B.1.617.2 (left panels) or Omicron BA.1 lineage (right panels) are shown in red.

In order to evaluate neutralizing activity against variants of SARS-CoV-2, VeroE6-TMPRSS2 cells were infected with WT SARS-CoV-2, Delta (B.1.617.2), or Omicron (BA.1). We counted the number of wells with CPE at 4 days post-infection and calculated the half maximal tissue culture infectious dose (TCID50). Although NIBIC-71 inhibited viral entry for WT SARS-CoV-2 and Delta, there was low inhibitory activity against Omicron (Fig. 5A). On the other hand, 7G7/κ had lower neutralizing activity against Delta than WT and did not have activity against Omicron (Fig. 5B). To explain these differences, we checked whether the mutation points in the S proteins of Delta and Omicron variants are overlapped with the binding regions of the two mAbs, based on our cryo-EM structures. The binding mode of NIBIC-71 is not affected by the L452R and T478K mutations in Delta variant (Fig. 5C, left panel), while many mutation points in Omicron variant (K417N, G446S, S477N, Q493R, G496S, Q498R, N501Y, and Y505H) are overlapped with the epitope of NIBIC-71 (Fig. 5C, right panel). T19R in Delta variant may affect the binding of 7G7/κ (Fig. 5D, left panel), and deletion of 142–144 residues and Y145D mutation in Omicron variant seem to transform the surface where CDR of 7G7/κ contacts (Fig. 5D, right panel).

## Discussion

In this study, we successfully developed 2 SARS-CoV-2 neutralizing mAbs with different mechanisms using our method involving PBMCs derived from individuals who recovered from WT SARS-CoV-2 infection. The purpose was to identify novel antigen regions in the S protein for obtaining safe, cross-reactive neutralizing mAbs by clarifying the mechanism of action for NIBIC-71 and 7G7. We identified the target regions of these mAbs and evaluated their neutralizing activity in vitro and in vivo. Furthermore, we analyzed the inhibition of S protein–ACE2 binding and S1/2 cleavage, which are both important events for SARS-CoV-2 entry. Our results suggested that S1/2 cleavage inhibition was useful as target of neutralizing mAb against SARS-CoV-2 equivalent to the blockade of S protein-ACE2 binding. We have shown that our method can produce neutralizing mAbs with various mechanisms.

The previously reported SARS-CoV neutralizing mAb S309 (sotrovimab) has cross-reactivity for WT SARS-CoV-2, Delta, and Omicron BA.1 lineage but not for BA.2 lineage^14,16,17^. LY-CoV1404 (bebtelovimab), which binds within the RBD and is affective against many VOCs including BA.2, has been reported to have the greatest range of neutralizing activity^18^. In summary, these reports suggested that cross-reactive mAbs could be obtained with a SHM rate of 2–5%. Therefore, we also aimed to isolate neutralizing mAbs with cross-reactivity. The SHM rates of our mAbs were in this range (Table 1). However, NIBIC-71 and 7G7/κ did not have neutralizing activity against Omicron BA.1 lineage. One possible reason is that the number of days after infection in convalescent individuals used for this study was not sufficient to isolate neutralizing mAbs with cross-reactivity. LY-CoV1404 was isolated from PBMCs derived from a donor approximately 60 days after the onset of COVID-19^18^. In addition, it has been reported that neutralizing mAbs with cross-reactivity against influenza 1H1N and 1H5N were isolated from healthy individuals at 28 days after the second vaccination with the pandemic H1N1 2009 vaccine; these individuals had been previously vaccinated with the seasonal vaccine 4–160 days before receiving the pandemic H1N1 2009 vaccine^19^. Moreover, a previous study of human immunodeficiency virus (HIV)-neutralizing mAbs reported that a greater breadth of neutralizing mAbs can be obtained from a blood sample collected from a patient at 119 weeks after HIV infection than at 59 weeks^20^. These findings suggest that it is desirable to use blood samples from individuals who have been exposed to antigens at least 4 weeks prior in order to obtain neutralizing mAbs with a broad range of neutralizing activity. Currently, since multiple vaccinations against SARS-CoV-2 infection are progressing all over the world, it is expected that cross-reactive neutralizing mAbs can be obtained from immunized healthy volunteers.

7G7/κ interacts with the NTD (Fig. 3D) and suppressed S1/2 cleavage, resulting in evasion of viral entry (Fig. 3E,G). Although we used whole mAbs for cryo-EM structural analysis, only the Fab regions could be observed because of the highly flexible Fc regions. As the three NTD epitopes of 7G7/κ in the S protein trimer are distant from each other, each Fab bound to the S protein should be derived from different mAbs, keeping the other unbound Fab free and flexible. Therefore, the other Fab from the same mAb molecule can catch another S protein trimer. In the presence of excess 7G7/κ compared to the S protein, 7G7/κ could gather the S proteins, which may limit the access of furin protease to the cleavage site. In contrast, the two Fabs of NIBIC-71 bound to the up-RBDs are very close to each other and must be derived from the same mAb molecule. The mAb binding to remaining “unoccupied” RBD could also bridge two S protein trimers, but the conformation change to up-RBD should be necessary. Mixing of the mAbs with S trimers in 1:1 molar ratio in the cryo-EM experiments may have suppressed their aggregations, leading to the successful cryo-EM structural analysis. In summary, 7G7/κ recognizes the NTD, which are distant from each other in the S protein trimer and has three bridging points per one S protein. In contrast, one NIBIC-71 must be occupying the two RBDs because of their vicinity in the up conformation and therefore has only one remaining bridging point per one S protein. The distance between the epitopes in the S trimer could be key to regulate the aggregation of S trimers that modulate the activity of the proteases.

Many mutations in the S protein have been found in the Delta and Omicron variants^12,14^. Since NIBIC-71 binds to the RBD (Fig. 3B,C) and inhibits viral entry for WT and Delta (Fig. 5A), the mutations in the S protein of Delta do not affect the access of NIBIC-71 to the S protein, as the cryo-EM structure shows (Fig. 5C, left panel). In contrast, NIBIC-71 shows a complete loss of neutralizing activity against Omicron BA.1 lineage because of the many mutations in RBD (Fig. 5C, right panel). In addition, 7G7/κ had a lower inhibitory effect on Delta infection than WT infection and no effect on Omicron infection (Fig. 5B) because of the fewer but severe mutations in NTD (Fig. 5D). Furthermore, it has been reported that with the exception of bebtelovimab, most neutralizing mAbs approved as therapeutic agents have significantly lower neutralizing activity against Omicron variant lineages BA.1 and BA.2 compared with WT SARS-CoV-2^17^. These results demonstrate that the reported epitopes in the RBD and NTD may not be suitable as antigens for obtaining neutralizing mAbs against VOCs. Compared to the S protein of WT SARS-CoV-2, the Omicron BA.1 lineage had 3 amino acid (aa) mutations, 6 aa deletions, and 3 aa insertions in the NTD; 15 aa mutations in the RBD; 5 aa mutations in the SD; and 6 aa mutations in S2^14^. Since the SD had the fewest mutations in the S protein and some neutralizing mAbs targeting the NTD result in ADE, the SD is likely to be the most useful and safest novel target antigen for neutralizing mAbs. In addition, it is difficult to obtain neutralizing mAbs against various VOCs from volunteers who have experienced WT SARS-CoV-2 infection. Therefore, it is a priority to obtain a neutralizing mAb targeting the SD with cross-reactivity against emerging variants including Omicron from individuals who had been infected with Omicron more than 4 weeks after recovery from infection.

In conclusion, the one-to-one binding of NIBIC-71 to homotrimeric S protein via the RBD directly inhibits S protein–ACE2 binding. On the other hand, the binding of one molecule of 7G7 to two molecules of homotrimeric S protein, which suppresses access of furin to the S1/S2 cleavage site of S protein by aggregating S proteins, shows an indirect inhibitory effect (Fig. S3). Moreover, we found that blocking S1/2 cleavage suppresses infection as much as inhibition of S protein–ACE2 binding. Our mAbs had low cross-reactivity and were not able to inhibit Omicron BA.1 infection. However, it might be possible to obtain a greater range of neutralizing mAbs using our method with memory B cells derived from convalescent individuals more than 4 weeks after the onset of Omicron infection or healthy volunteers who have been vaccinated multiple times. This system, which enables the rapid isolation and analysis of functional mAbs, facilitates the elucidation of the mechanism of infection and the development of novel therapeutic strategies.

## Methods

### Virus

WT SARS-CoV-2 (2019-nCoV/Japan/TY/WK-521/2020), Delta (hCoV-19/Japan/TY11-927/2021) and Omicron (hCoV-19/Japan/TY38-873/2021) were employed.

### Cell culture

LCLs were cultured in LCL medium based on RPMI1640 (Nacalai Tesque) as described previously^21^. Sf9 cells were cultured in Sf-900TM II SFM (Thermo Fisher Scientific) at 28 °C. Expi293F cells were cultured in Expi293 Expression Medium in 8% CO_2_ at 37 °C. VeroE6-TMPRSS2 cells were cultured in Dulbecco’s modified Eagle’s medium (DMEM) (Nacalai Tesque) supplemented with 10% fetal bovine serum (FBS) (Merck) and streptomycin/penicillin (Nacalai Tesque).

### Purification of recombinant S proteins

The extracellular domain (ECD) (aa 13–1208) of the S protein–encoding gene was synthesized by Eurofins Genomics after codon optimization. The ECD, S1 (aa 13–685), S2 (aa 686–1208), NTD (aa 13–318), RBD (aa 319–541), NTD + RBD (aa 13–541), S1 RBD-deletion mutant (ΔRBD) (aa 13–318, aa 541–685), and SD (aa 541–685) were expressed (Thermo Fisher Scientific). In brief, truncated S proteins containing a *Drosophila* immunoglobulin heavy chain binding protein (BiP) secretion signal peptide, C-terminal flag, and 6 × His tags were amplified by PCR. They were inserted into the pFastBac1 vector and transformed into DH10Bac *Escherichia coli* competent cells (Thermo Fisher Scientific). Bacmids were extracted and subsequently transfected into Sf9 cells. Recombinant viruses were collected from the Sf9 supernatant and further multiplied to generate high-titer virus stock. Sf9 cells were infected with the high-titer virus stock to secrete recombinant proteins into culture medium. Recombinant proteins were purified from the supernatant using TALON Metal Affinity Resin (Takara Bio).

### Human blood samples

Blood samples originated from 2 convalescent volunteers who had been infected with SARS-CoV-2 in Japan in 2020. Ethics approval for the study was obtained from the institutional review board of the National Institutes of Biomedical Innovation, Health and Nutrition (approval number 198). Written informed consent was obtained from all participants. The study was performed in accordance with the guidelines of the Declaration of Helsinki.

### Human mAb cloning

Human mAb cloning was performed as described previously^21^. In brief, PBMCs were collected from blood samples. IgM + B cells were depleted from PBMCs. The remaining cells were suspended in LCL medium with EBV in round-bottomed 96-well plates for 2 weeks of culture; they transformed into LCLs. mAb sequences were determined with BCR amplicon sequencing. Deduction of antibody amino acid sequences was performed with LCMS. Next, IgH and IgL cDNA were obtained using nested PCR and subsequently subcloned into pQEIP and pGEFIN vectors.

### BCR amplicon sequencing

A cDNA library was made using reverse transcriptase from total RNA isolated from each LCL using a SMART cDNA Library Construction Kit (Takara Bio). Nested PCR was performed using previously described primers^21^. Afterwards, Illumina MiSeq paired-end sequencing was performed. Datasets were analyzed with the immunoinformatic tool MiXCR (v3.0.13), which included RepSeq IO (version 1.3.4), MiLib (version 1.3.4), and Built-in V/D/J library (version 1.6) to obtain the sequences of the VH and VL genes^22,23^.

### Deduction of antibody amino acid sequence and subclass with LCMS

To concentrate S protein–specific mAbs in the LCL supernatant, S protein binding beads were prepared; 10 µg of recombinant S ECD protein was dialyzed with boric acid buffer (50 mM, pH 8.5) using the Mini Dialysis kit (Cytiva). At the same time, 100 µL of N-hydroxy succinimide (NHS)-Activated Magnetic Beads (Thermo Fisher Scientific) were washed with 350 µL of ice-cold 1 mM HCl. This mixture was added to 100 µL of recombinant S ECD protein and then rotated for 2 h at room temperature. Subsequently, the magnetic beads were washed with 350 µL of glycine (0.1 M, pH 2.0) twice, suspended in 350 µL of ethanolamine (3 M, pH 9.0), and rotated for 90 min at room temperature. After rotation, the beads were washed with 350 µL of distilled water 3 times. The beads were finally suspended in 100 µL of LCL medium. Next, 10 µL of the S protein binding beads were added to 50 µL of the LCL supernatant and rotated overnight at 4 °C. The binding beads were washed with 500 µL of wash buffer (ice-cold 50 mM Tris–HCl pH 8.0, 125 mM NaCl) 5 times. Next, 30 µL of Sample Buffer Solution (Nacalai Tesque) was added to the beads. The samples were boiled for 10 min at 100 °C and were separated with SDS-PAGE. Concentrated S protein–specific antibodies were fragmented to peptides with in-gel digestion. They were characterized using LCMS Orbitrap Fusion Lumos (Thermo Fisher Scientific) as described previously^24^.

### Construction of 7G7-IgG1κ antibody expression vectors

To construct 7G7/κ (IgG1/Igκ) antibody expression vectors, a constant kappa light chain (Cκ) gene was merged with the VL gene of 7G7/λ (IgG1/Igλ2). Briefly, the VL gene of 7G7/λ and the Cκ gene were amplified with PCR. An expression vector sequence was also amplified along with the Cκ gene for cloning. Approximately 20-nucleotide overlaps were added to both the 5′ and 3′ ends of the PCR products. The PCR products were then assembled using the NEBuilder HiFi DNA Assembly Master Mix (New England BioLabs).

### Analysis of immunoglobulin genes

The rate of mutation was examined by comparing our mAb gene sequence with the sequence of the germline antibody as described previously^21^.

### Purification of recombinant mAbs

Purification of recombinant mAbs was performed using the Expi293 Expression system (Thermo Fisher Scientific) as described previously^21,25^. In brief, Expi293F cells were cotransfected with a mixture of IgH and IgL expression vectors and cultured for 7 days. The culture supernatants were loaded onto HiTrap Protein G HP Columns (Cytiva). Recombinant mAbs were eluted from the columns and dialyzed against PBS buffer.

### ELISA

ELISA was conducted as described previously^21,25^. The reactivity of LCL supernatants and recombinant mAbs were determined using 50 ng of recombinant truncated S proteins using ELISA. The previously constructed human mAb 8A7 was used as a negative control for the ELISA experiment^21^. The concentration of IgG was measured relative to human IgG (SouthernBiotech) with ELISA.

### SPR analysis

SPR analyses for the recombinant antibodies NIBIC-71, 7G7/λ, and 7G7/κ were carried out using a Biacore T200 instrument (Cytiva) as previously described^25^. Briefly, anti–human IgG (Fc) antibodies were immobilized on a Series S Sensor Chip CM5 (Cytiva) using the Human Antibody Capture Kit (Cytiva) and the Amine Coupling Kit (Cytiva). The human mAb 8A7 was used as a negative control in SPR analyses. Each antibody was captured at approximately 150 response units (RUs). SPR sensorgrams were obtained by injecting twofold serial dilutions of SARS-CoV-2 S protein (ECD, His and Flag Tag) (GenScript) ranging from 80 to 0.625 nM in PBS-T. The experimental parameters were as follows: temperature, 25 °C; flow rate, 30 µL/min; contact time, 240 s; and dissociation time, 900 s. The SPR sensorgrams of each antibody were analyzed using Biacore T200 Evaluation Software version 2.0 (Cytiva).

### CPE assay

To screen for LCL supernatants that contained S protein–specific antibodies, VeroE6-TMPRSS2 cells were seeded at 1 × 10^4^ cells/well in 96-well plates and cultured overnight. WT SARS-CoV-2 was incubated in LCL supernatant diluted 5-flod in fresh LCL medium or only LCL medium as a negative control at room temperature for 1 h. Incubated SARS-CoV-2 was added to each well, which was then incubated at 37 °C in 5% CO_2_ for 1 h. After incubation, these cells were replaced with fresh DMEM supplemented with 1% FBS and were infected for 3 days. The infected cells were fixed by formaldehyde neutral buffer solution (Kishida). The number of cells with CPE was counted in each well using ImageJ software.

### Cryo-EM specimen preparation and data collection

S protein trimer with D614G mutation was prepared as described previously^26^ and mixed with 1:1 molar ratio of NIBIC-71 and 7G7/κ solutions at a final concentration of 0.5 mg/mL of the S protein trimer. Quantifoil grids (R1.2/1.3 Cu 200 mesh) were glow-discharged using a JEC-3000FC sputter coater (JEOL) at 20 mA for 20 s. After 3 mL of the complex solutions were applied, the grids were blotted with a force of –10 and a time of 2 s in a Vitrobot Mark IV chamber (Thermo) equilibrated at 4 °C and 100% humidity, and then immediately plunged into liquid ethane. The grids were stored in liquid nitrogen. All cryo-EM image datasets were acquired using SeriaIEM^27^, yoneoLocr^28^, and a JEM-3300 (CRYO ARM™ 300 II, JEOL) operated at 300 kV with a K3 direct electron detector (Gatan, Inc.) in CDS mode. The W-type in-column energy filter was operated with a slit width of 20 eV for zero-loss imaging. The nominal magnification was 60,000×, corresponding to 0.86 Å per pixel. Defocus varied between − 0.5 and − 2.0 mm. Each movie was fractionated into 60 frames with a total dose of 60 e^−^/Å^2^.

### Cryo-EM image processing

The images were processed using RELION 4.0^29^. Movies were motion corrected using MotionCor2^30^, and the contrast transfer functions (CTFs) were estimated using CTFFIND 4.1^31^. Micrographs whose CTF max resolutions were beyond 5 Å were selected. 3D template-based autopicking was performed for all images, and the particles were extracted with 4 × binning, which were subjected to two rounds of 2D classification. An initial model was generated and used as a reference for the following 3D classification. Reference-based 3D classification (into 4 classes) was performed, and the selected particles were re-extracted with binning (box size from 512 to 336 pixel for the Spike + NIBIC-71 dataset, and from 480 to 320 pixel for the Spike + 7G7/κ dataset). Another round of 2D classification was conducted, and selected particles were subjected to 3D refinement, soft mask generation, and postprocessing. Focused 3D classification without alignment was performed with the antibody regions masked to eliminate particles without binding the antibodies. For the Spike + NIBIC-71 dataset, selected particles were re-extracted again with smaller binning (box size from 512 to 420 pixel). The particles were subjected to another round of 3D refinement, soft mask generation, postprocessing, CTF refinement, Bayesian polishing, 3D refinement, soft mask generation, and postprocessing. Finally, another round of CTF refinement, 3D refinement, and postprocessing were performed with optics groups divided (500 micrographs per group). The final map resolutions (FSC = 0.143) were 2.64 Å and 2.96 Å in the Spike + NIBIC-71 and Spike + 7G7/κ datasets, respectively.

Homology models of NIBIC-71 and 7G7/κ Fabs were generated by SWISS-MODEL^32^. The atomic model of S protein^26^ and the homology models of the Fabs were manually fitted into the density by using UCSF Chimera^33^ and Coot^34^. Figures were prepared by using ChimeraX^35^ and PyMOL (Schrödinger, LLC). The parameters are summarized in Table 3.

### In vitro neutralization assay

To evaluate neutralizing activity of NIBIC-71 and 7G7/κ using NanoLuc Luciferase technology, VeroE6-TMPRSS2 cells were seeded at 1 × 10^4^ cells/well in 96-well plates and cultured overnight before infection. 10^3^ TCID50/well of NanoLuc Luciferase introduced WT SARS-CoV-2 were mixed with serially diluted NIBIC-71, 7G7/κ, or N.C. mAb (8A7) at room temperature for 1 h. The mixture was then added to each well and incubated at 37 °C in 5% CO_2_ for 1 h. Following incubation, the medium was exchanged to fresh DMEM supplemented with 1% FBS. Infected cells were dissolved with passive lysis buffer (Promega). The lysates underwent reaction with Nano-Glo Luciferase Assay Substrate in the Nano-Glo Luciferase Assay System (Promega). Luminescence intensity was detected with a luminometer. To measure TCID50, a total of 10^4^ VeroE6-TMPRSS2 cells per well were seeded into 96-well plates before infection in quadruplicate; recombinant mAb (10 μg/mL) was serially diluted at 1:3 to 0.001 µg/mL and incubated with 10^2^ TCID50 of WT SARS-CoV-2, Delta, or Omicron for 1 h at 37 °C. Next, 100 µL of each mixture was added to individual wells. After 4 days of incubation at 37 °C in 5% CO_2_, infected cells were fixed with formaldehyde neutral buffer solution and stained with 1% crystal violet (Nacalai Tesque). The number of wells with CPE was counted. TCID50 was calculated using the Spearman and Karber algorithm.^36^.

### S protein-ACE2 binding assay

Recombinant S ECD protein (50 ng) was incubated overnight at 4 °C in individual Nunc MaxiSorp™ flat-bottom wells (Thermo Fisher Scientific). A series of 1:3 dilutions was made by mixing each recombinant mAb with PBS from 10 to 0.001 µg/mL. After blocking with 100 µL of blocking buffer (1 × PBS, 2% BSA, 0.05% NaN_3_) at room temperature for 1 h, each dilution or PBS (N.C.) (50 µL) was added to wells and incubated at room temperature for 1 h. Following washing with PBS-T (1 × PBS, 1% Tween) 3 times, 100 ng of recombinant human ACE-2 His-tag biotinylated protein (R&D Systems) was incubated at room temperature for 1 h in each well. Next, horseradish peroxidase (HRP)-conjugated streptavidin (Thermo Fisher Scientific) was diluted at 1:10,000, added to each well at 50 µL/well, and incubated at room temperature for 1 h. One-step™ Ultra TMB-ELISA Substrate Solution (Thermo Fisher Scientific) (25 µL/well) was added to each well at room temperature. The reaction was quenched with 25 µL/well of STOP solution (2N H_2_SO_4_). Absorbance was read at 450 nm using an absorbance plate reader.

### S1/S2 cleavage assay

SARS-CoV-2 S protein (ECD, His and Flag Tag) (GenScript) (50 ng) was diluted in reaction buffer (20 mM HEPES, 1 mM CaCl_2_, 0.2 mM 2-mercaptoethanol, 0.1% Triton X-100) and was incubated overnight at 4 °C in individual Nunc MaxiSorp™ flat-bottom wells (Thermo Fisher Scientific). A series of 1:3 dilutions was made by mixing each recombinant mAb with reaction buffer from 10 to 0.001 µg/mL. After blocking with 100 µL of blocking buffer (1 × PBS, 2% BSA, 0.05% NaN_3_) at room temperature for 1 h, each dilution or reaction buffer (N.C.) (50 µL) was added to wells and incubated at room temperature for 1 h. After blocking and washing with reaction buffer 3 times, 1 unit of furin (New England BioLabs) or only reaction buffer without furin was added to each well up to total 20 µL at room temperature for 24 h. Next, 50 ng/mL of 6x-His Tag Monoclonal Antibody-HRP (HIS.H8) (Thermo Fisher Scientific) was added to each well at temperature for 1 h. After washing with PBS-T 3 times, One-step™ Ultra TMB-ELISA Substrate Solution (Thermo Fisher Scientific) (25 µL/well) was added to each well at room temperature. The reaction was quenched with 25 µL/well of STOP solution (2N H_2_SO_4_). Absorbance was read at 450 nm using an absorbance plate reader. Inhibition efficiency was calculated by dividing the absorbance of the wells including S protein, each diluted antibody and furin against the absorbance of only S protein.

### In vivo infectious experiment

Male 3-week-old Syrian hamsters were purchased from SLC. Firstly, hamsters were given 4 mg/kg of recombinant mAbs intravenously. Following inhalational isoflurane anesthesia (MSD Animal Health), they subsequently were infected with 10^3^ TCID50/20 µL of WT SARS-CoV-2 via the nasal route. Body weight and rectal temperature were monitored daily up to 4 days post-infection. Next, lung tissue was collected from hamsters infected at 4 days post-infection. The tissue was separated for viral copy count, measurement of TCID50, and histological analysis.

Regarding the number of viral copies, 1 mL of TRIzol Reagent (Thermo Fisher Scientific) was added to lung tissue. The tissue was physically disrupted using a bead-type disruptor. Total RNA was extracted from disrupted tissues. The number of viral copies was measured using the real-time one-step RT-PCR method and a TaqMan probe according to the Manual for the Detection of Pathogen 2019-nCoV (https://www.niid.go.jp/niid/images/epi/corona/2019-nCoVmanual20200217-en.pdf, National Institute of Infectious Diseases, Japan).

For TCID50 measurement, the other part of lung tissue was weighed and 0.5 mL of PBS was added. The tissue was physically disrupted using a bead-type disruptor. The supernatant was collected after centrifugation, diluted 500-fold, and further serially diluted at 1:10 with PBS. Next, 10^4^ VeroE6-TMPRSS2 cells were seeded in 100 µL of cell culture medium per well on a 96-well plate and cultured overnight. Diluted supernatant was added to each well, which was then incubated in 5% CO_2_ at 37 °C for 4 days. The number of wells with CPE was counted, which was presented as TCID50.

For histological analysis, the remaining tissue was fixed for at least 7 days in formaldehyde neutral buffer solution. Tissues were embedded in paraffin. The paraffin blocks were cut into 3-µm-thick sections. Sections were stained as described previously^37^. To detect SARS-CoV-2 nucleocapsid protein in immunohistochemistry analyses, tissue sections were incubated with SARS-CoV-2 nucleocapsid protein mAb (Clone 1035111) (R&D Systems) and then Histofine® MAC-PO (Multi) (Nichirei Biosciences).

### Quantification of infiltrated area

To measure the area of infiltrated cells and lung epithelial cells, 5 sections were obtained from the HE-staining sections of each hamster. These sections were converted to gray-scale. The areas of HE-staining cells including epithelial cells were defined as infiltrated area, and were calculated by ImageJ software^15^.

## Supplementary Information

Supplementary Information 1.

Supplementary Information 2.

Supplementary Information 3.

## Data Availability

Cryo-EM density maps are available at the Electron microscopy Data Bank (EMDB) with accession codes: EMD-34031 (Spike + NIBIC-71) and EMD-34032 (Spike + 7G7/κ). Additional cryo-EM data supporting this study are available from K.N. on reasonable request.
