# Supplementary material for: Human antibody recognition and neutralization mode on the NTD and RBD domains of SARS-CoV-2 spike protein
Source: Sci Rep. 2022 Nov 22;12:20120. doi: 10.1038/s41598-022-24730-4 (PMC9684487; doi:10.1038/s41598-022-24730-4)
Supplement: Supplementary file 1 — Supplementary Information 1. [file 41598_2022_24730_MOESM1_ESM.pdf]

Fig. S1

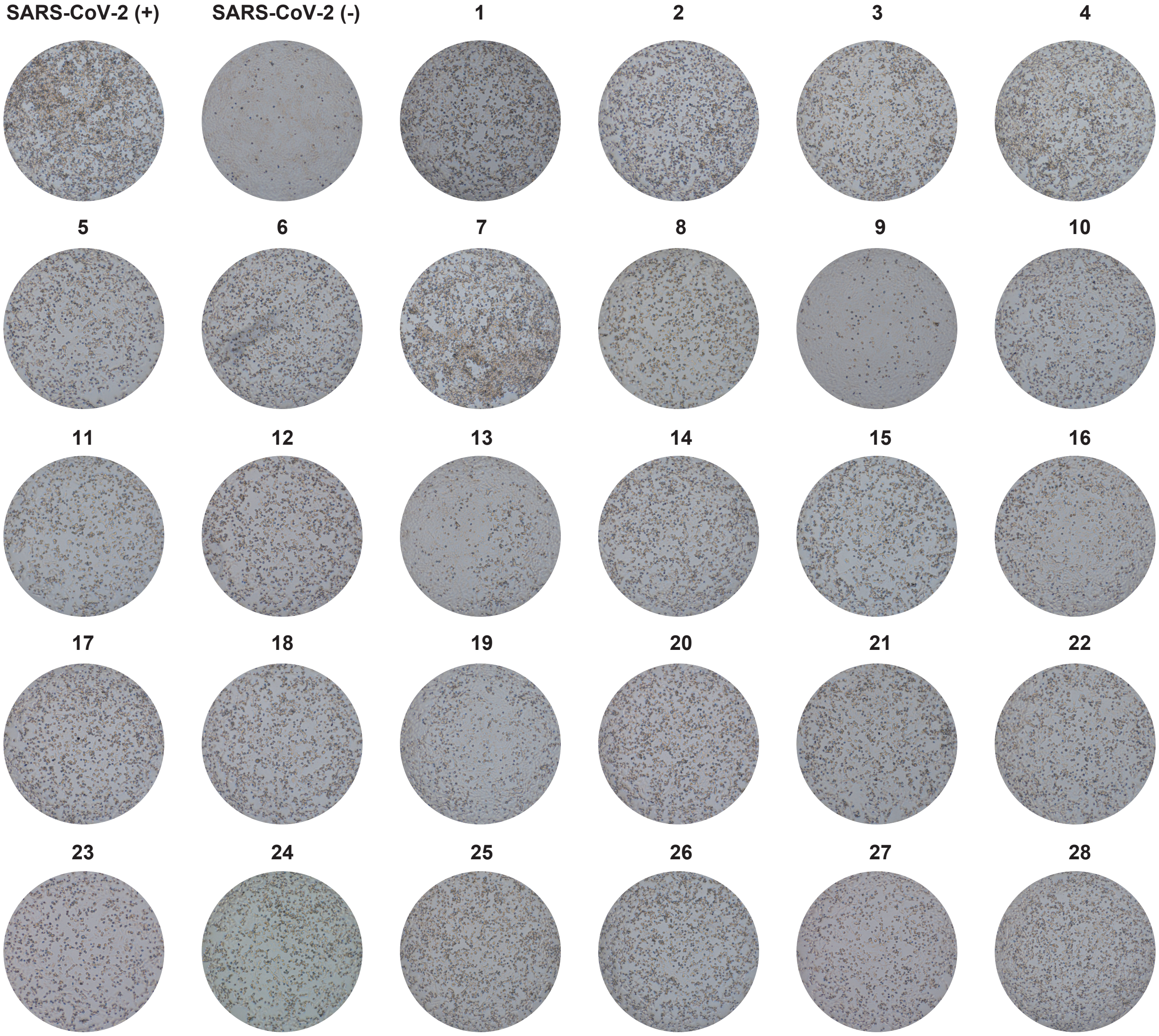

Supplementary Figure 1. Screening of LCL supernatants including neutralizing mAb by CPE assay

VeroE6-TMPRSS2 cells were infected with WT SARS-CoV-2 pre-incubated with each 5-fold diluted LCL supernatant (Clone ID:1~28) or only LCL medium or without virus for 4 days. These photos were converted to gray-scale and counted cells with CPE by imageJ software.
