# Supplementary material for: Human antibody recognition and neutralization mode on the NTD and RBD domains of SARS-CoV-2 spike protein
Source: Sci Rep. 2022 Nov 22;12:20120. doi: 10.1038/s41598-022-24730-4 (PMC9684487; doi:10.1038/s41598-022-24730-4)
Supplement: Supplementary file 2 — Supplementary Information 2. [file 41598_2022_24730_MOESM2_ESM.pdf]

Fig. S2

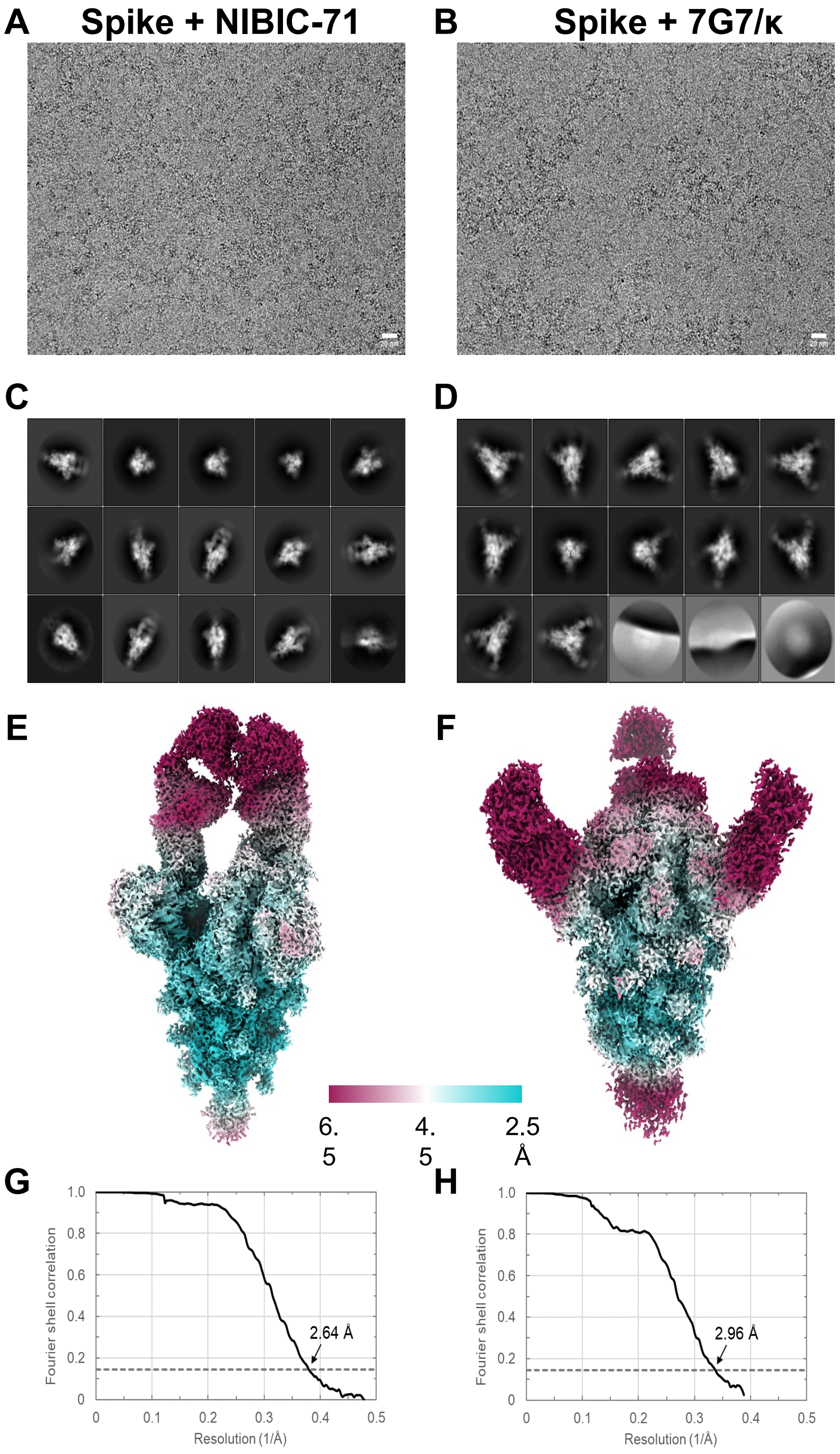

Supplementary Figure 2. Cryo-EM analysis of the homotrimeric S protein complexed with NIBIC-71 or 7G7/κ

A and B, Typical motion-corrected micrographs (x 600,000). C and D, Top 15 2D class averages aligned in the descending order of particle numbers from left to right and top to bottom. E and F, Final sharpened maps shown in Fig. 3C and D but colored by the local resolution distributions as in the color bar. G and H, The FSC curves for the final map. The dashed lines indicate the FSC = 0.143 criterion.
