# Supplementary material for: Human antibody recognition and neutralization mode on the NTD and RBD domains of SARS-CoV-2 spike protein
Source: Sci Rep. 2022 Nov 22;12:20120. doi: 10.1038/s41598-022-24730-4 (PMC9684487; doi:10.1038/s41598-022-24730-4)
Supplement: Supplementary file 3 — Supplementary Information 3. [file 41598_2022_24730_MOESM3_ESM.pdf]

Fig. S3

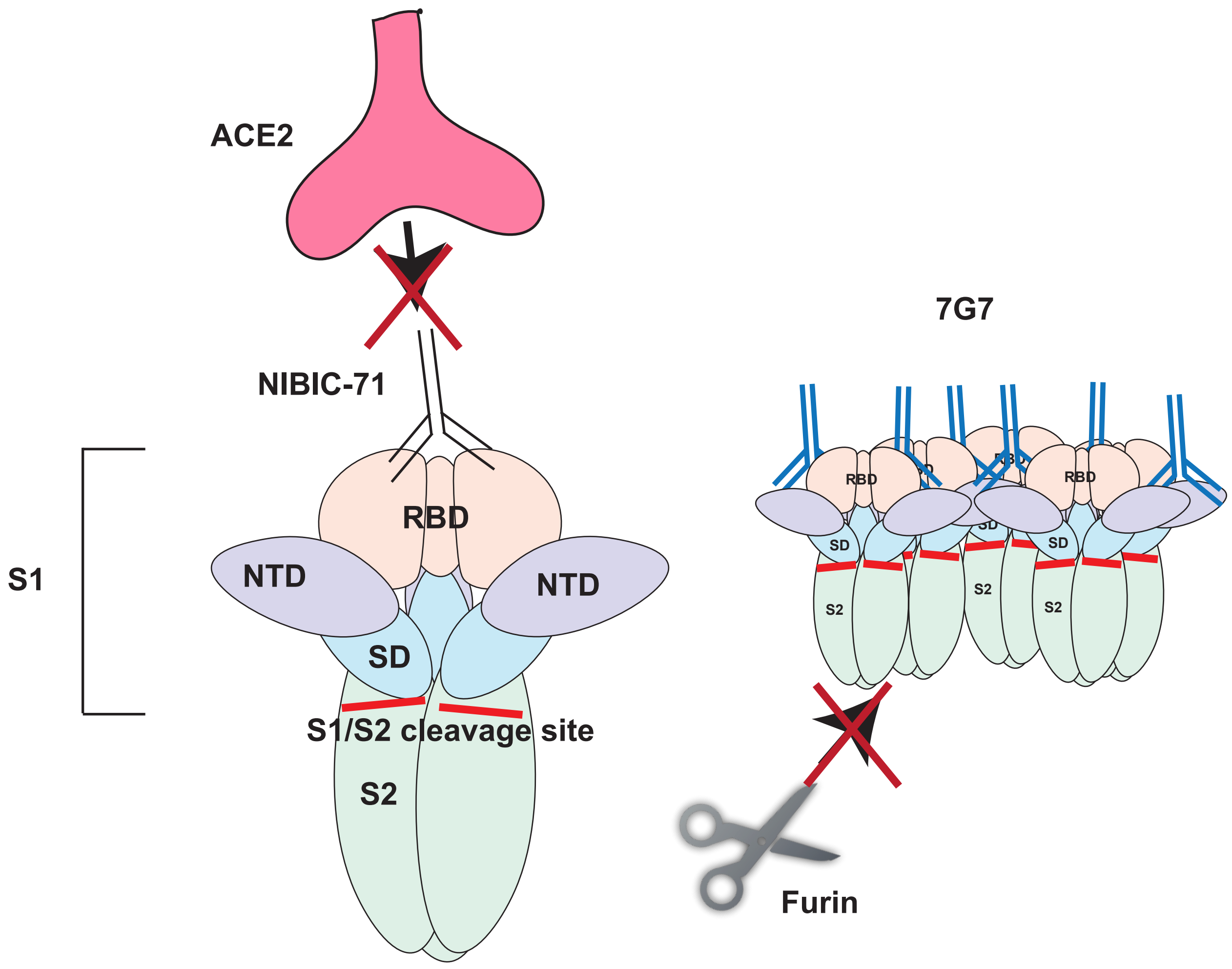

Supplementary Figure 3. Inhibitory models of NIBIC-71 and 7G7 binding to the S protein

NIBIC-71 binds to the RBD (left), resulting in direct inhibitory effect on S protein-ACE2 binding.

On the otherhand, 7G7 interacted with the NTD (right), causing indirect inhibitory effect on access of furin to the S1/S2 cleavage site
